# Spinal cord quadruple misfolded proteins in chronic traumatic encephalopathy

**DOI:** 10.1002/alz70855_100291

**Published:** 2025-12-23

**Authors:** Hidetomo Tanaka, Lauren E. Black, Shelley L. Forrest, Carmela Tartaglia, Mozhgan Khodadadi, Nusrat Sadia, Charles Tator, William Stewart, Gabor G. Kovacs

**Affiliations:** ^1^ Tanz Centre for Research in Neurodegenerative Disease and Department of Laboratory Medicine and Pathobiology, University of Toronto, Toronto, ON, Canada; ^2^ University of Glasgow, Glasgow, United Kingdom; ^3^ University of Toronto, Toronto, ON, Canada; ^4^ Canadian Concussion Centre, Toronto Western Hospital, University Health Network, Toronto, ON, Canada; ^5^ Canadian Concussion Centre, Toronto Western Hospital, Krembil Brain Institute, University Health Network, Toronto, ON, Canada; ^6^ Queen Elizabeth University Hospital, Glasgow, United Kingdom; ^7^ Department of Laboratory Medicine and Pathobiology, University of Toronto, Toronto, ON, Canada; ^8^ Tanz Centre for Research in Neurodegenerative Diseases, University of Toronto, Toronto, ON, Canada; ^9^ Krembil Brain Institute, Toronto, ON, Canada

## Abstract

**Background:**

Tau pathology in the brain associated with chronic traumatic encephalopathy (CTE) is well established, but details regarding protein pathology in the spinal cord remain unclear. Furthermore, CTE is frequently comorbid with other neurodegenerative diseases.

**Method:**

Spinal cords from 28 cases, including CTE (*n* = 14), Alzheimer's disease (AD, *n* = 6), one further case with repetitive head impacts but not CTE lesions in the brain and controls (*n* = 7; 4 with spinal stenosis), were examined using immunohistochemistry for *p*‐tau, TDP‐43, α‐synuclein, and A‐beta.

**Result:**

Misfolded protein deposition in the spinal cord was common in CTE: *p*‐tau (14/14, 100%), *p*‐TDP‐43 (9/14, 64%), Aβ (13/14, 93%), and α‐Syn (7/14, 50%). Quadruple misfolded protein deposition was observed in four CTE cases. Neuronal tau pathology was present in all CTE cases. Notably, prominent astrocytic tau pathology was observed in 12/14 cases (86%). However, no such pathology was observed in AD or control cases. *p*‐TDP‐43 pathology was frequently detected in the spinal cords of CTE cases. Four cases exhibited spinal cord‐specific *p*‐TDP‐43 inclusions without LATE/FTLD‐TDP. Aβ deposition was observed in most CTE spinal cords, with two spinal cord‐positive cases lacking cerebrum deposition, highlighting a brain‐spinal cord discrepancy. All seven CTE cases with α‐Syn pathology in the spinal cord had Lewy body pathology in the brain.

**Conclusion:**

Our findings demonstrate repetitive traumatic events may lead to tau pathology not only in the brain but also in the spinal cord. The prominent astrocytic tau pathology observed in CTE appears to be a unique feature, as it was not present in AD and control cases. The frequent co‐presence of tau with TDP‐43, Aβ, and α‐Syn pathologies suggests that repetitive traumatic events contribute to concomitant misfolded protein accumulation.

